# Supplementary material for: PTMNavigator: interactive visualization of differentially regulated post-translational modifications in cellular signaling pathways
Source: Nat Commun. 2025 Jan 8;16:510. doi: 10.1038/s41467-024-55533-y (PMC11711753; doi:10.1038/s41467-024-55533-y)
Supplement: Supplementary file 1 — Supplementary Information [file 41467_2024_55533_MOESM1_ESM.pdf]

Supplementary Information for

# PTMNavigator: Interactive Visualization of Differentially Regulated Post-Translational Modifications in Cellular Signaling Pathways

Julian Müller[1], Florian P. Bayer[1], Mathias Wilhelm[2], Maximilian G. Schuh[1†], Bernhard Kuster[1,3], and Matthew The[1]*

[1]Proteomics and Bioanalytics, School of Life Sciences, Technical University of Munich; 85354 Freising, Germany.

[2]Computational Mass Spectrometry, School of Life Sciences, Technical University of Munich; 85354 Freising, Germany.

[3]German Cancer Consortium (DKTK), Partner Site Munich, 80336 Munich, Germany.

[†]Present Address: Organic Chemistry II, School of Natural Sciences, Technical University of Munich; 85748 Garching, Germany.

*Corresponding author.

This PDF contains Supplementary Figures 1 to 10.

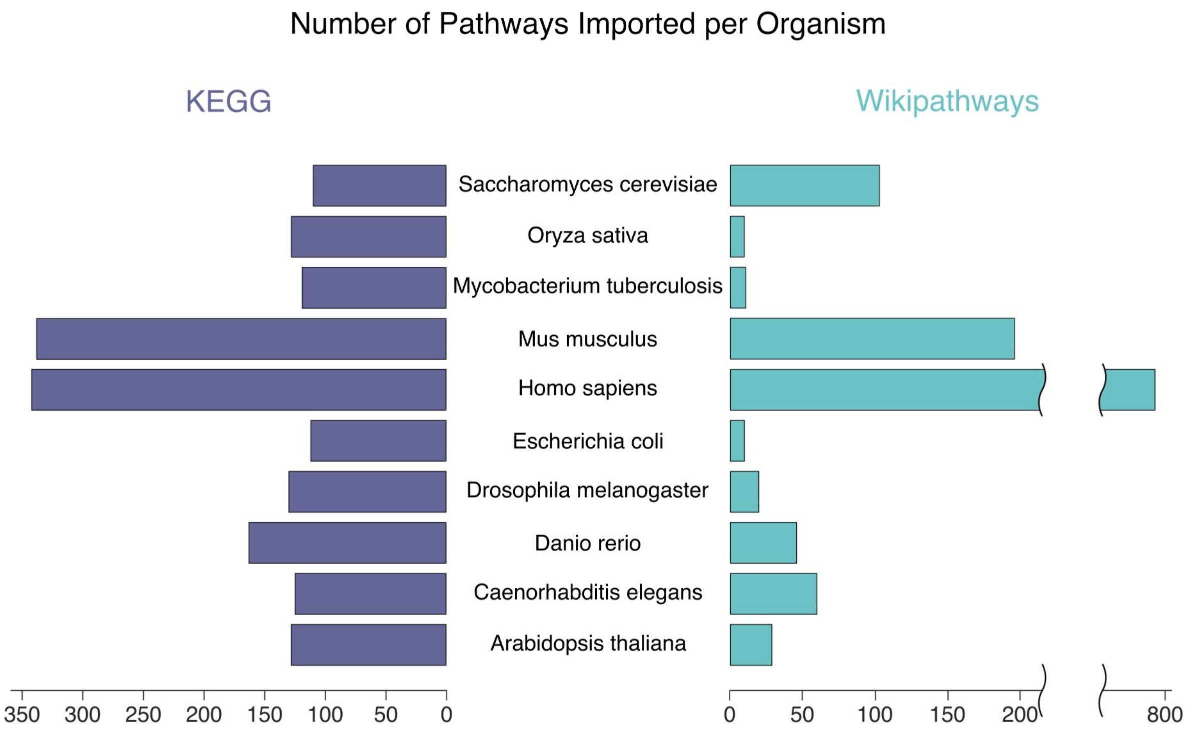


**Supplementary Figure 1. Number of pathways per organism and reference database that were imported into ProteomicsDB and are available in PTMNavigator.**

*Source data are provided as a Source Data file.*

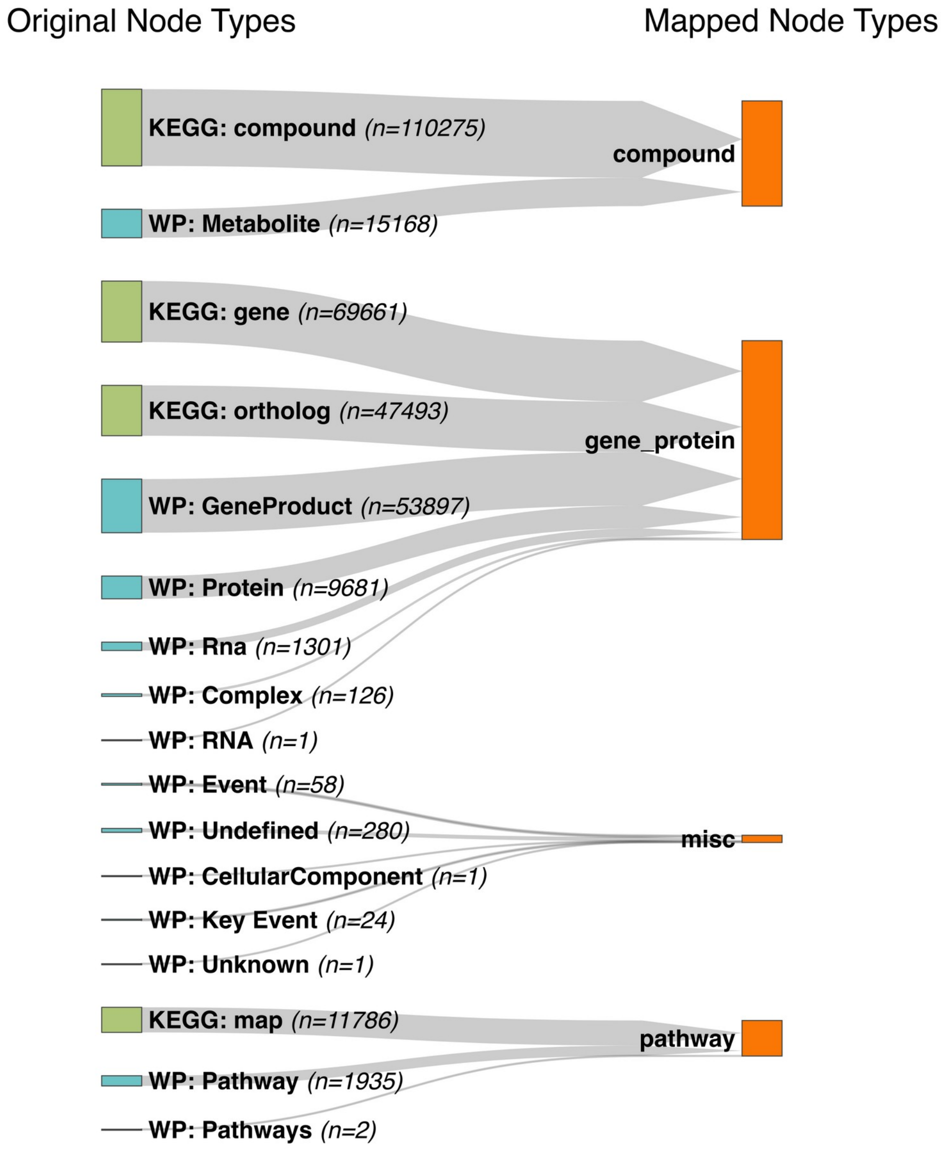


**Supplementary Figure 2. Mapping of Node Types from KEGG and WikiPathways to PTMNavigator.**

*On the left side all node types are listed that appear in the KEGG KGML or WikiPathways GPML files of the ten imported organisms. On the right side are the 4 node types that are defined in PTMNavigator. Source data are provided as a Source Data file.*

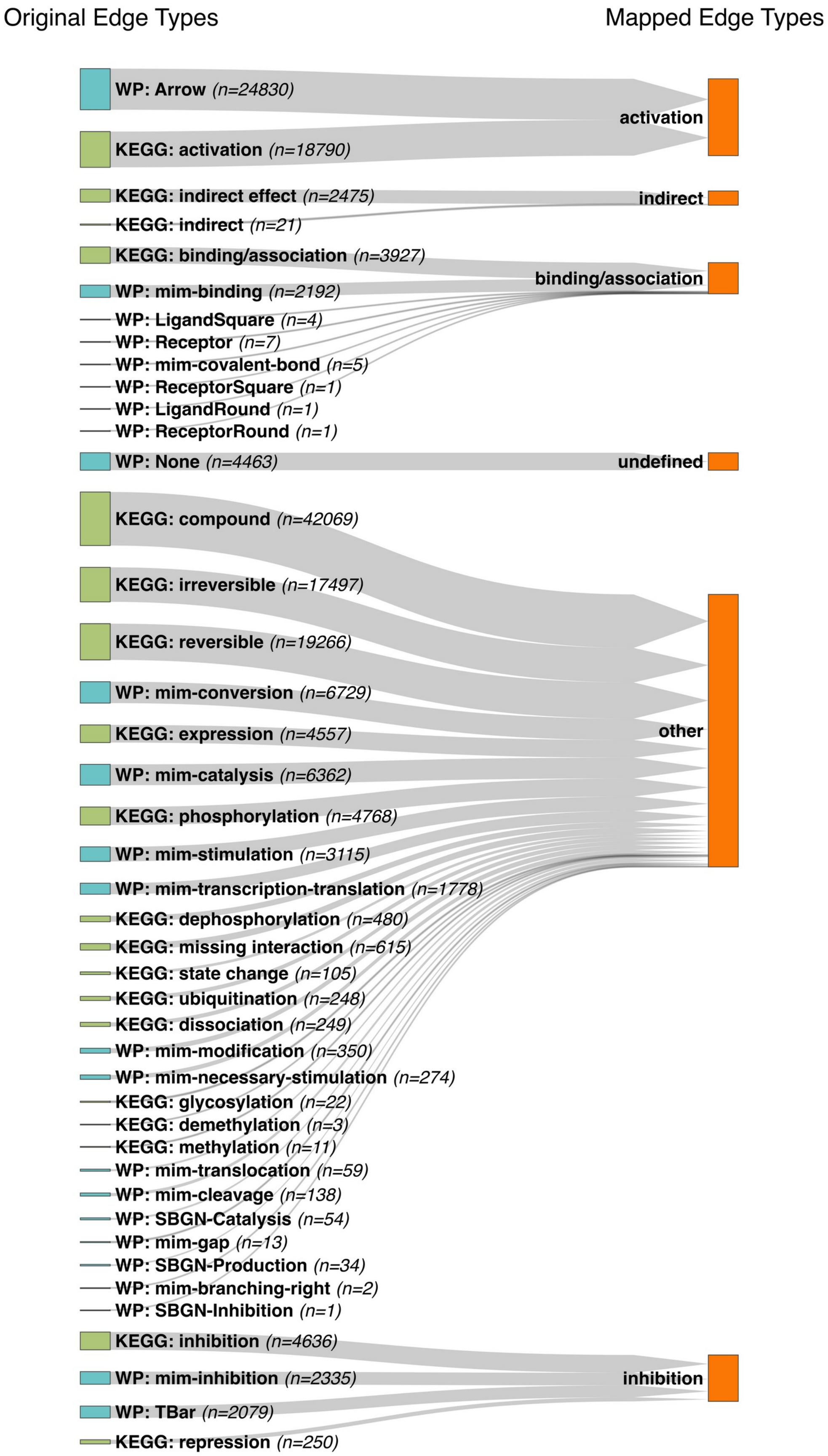

Original Edge Types
Mapped Edge Types
WP: Arrow (n=24830)
KEGG: activation (n=18790)
KEGG: indirect effect (n=2475)
KEGG: indirect (n=21)
KEGG: binding/association (n=3927)
WP: mim-binding (n=2192)
WP: LigandSquare (n=4)
WP: Receptor (n=7)
WP: mim-covalent-bond (n=5)
WP: ReceptorSquare (n=1)
WP: LigandRound (n=1)
WP: ReceptorRound (n=1)
WP: None (n=4463)
KEGG: compound (n=42069)
KEGG: irreversible (n=17497)
KEGG: reversible (n=19266)
WP: mim-conversion (n=6729)
KEGG: expression (n=4557)
WP: mim-catalysis (n=6362)
KEGG: phosphorylation (n=4768)
WP: mim-stimulation (n=3115)
WP: mim-transcription-translation (n=1778)
KEGG: dephosphorylation (n=480)
KEGG: missing interaction (n=615)
KEGG: state change (n=105)
KEGG: ubiquitination (n=248)
KEGG: dissociation (n=249)
WP: mim-modification (n=350)
WP: mim-necessary-stimulation (n=274)
KEGG: glycosylation (n=22)
KEGG: demethylation (n=3)
KEGG: methylation (n=11)
WP: mim-translocation (n=59)
WP: mim-cleavage (n=138)
WP: SBGN-Catalysis (n=54)
WP: mim-gap (n=13)
WP: SBGN-Production (n=34)
WP: mim-branching-right (n=2)
WP: SBGN-Inhibition (n=1)
KEGG: inhibition (n=4636)
WP: mim-inhibition (n=2335)
WP: TBar (n=2079)
KEGG: repression (n=250)
activation
indirect
binding/association
undefined
other
inhibition

**Supplementary Figure 3. Mapping of Edge Types from KEGG and WikiPathways to PTMNavigator.**

*On the left side all edge types are listed that appear in the KEGG KGML or WikiPathways GPML files of the ten imported organisms. The right side shows how they are mapped to the internal JSON representation in ProteomicsDB. Raw edge types are converted either to 'activation', 'inhibition', 'binding/association', 'indirect' or 'undefined'. The other edges are not converted and retain their original type (in the figure, these edge types are summarized as 'other'). Source data are provided as a Source Data file.*

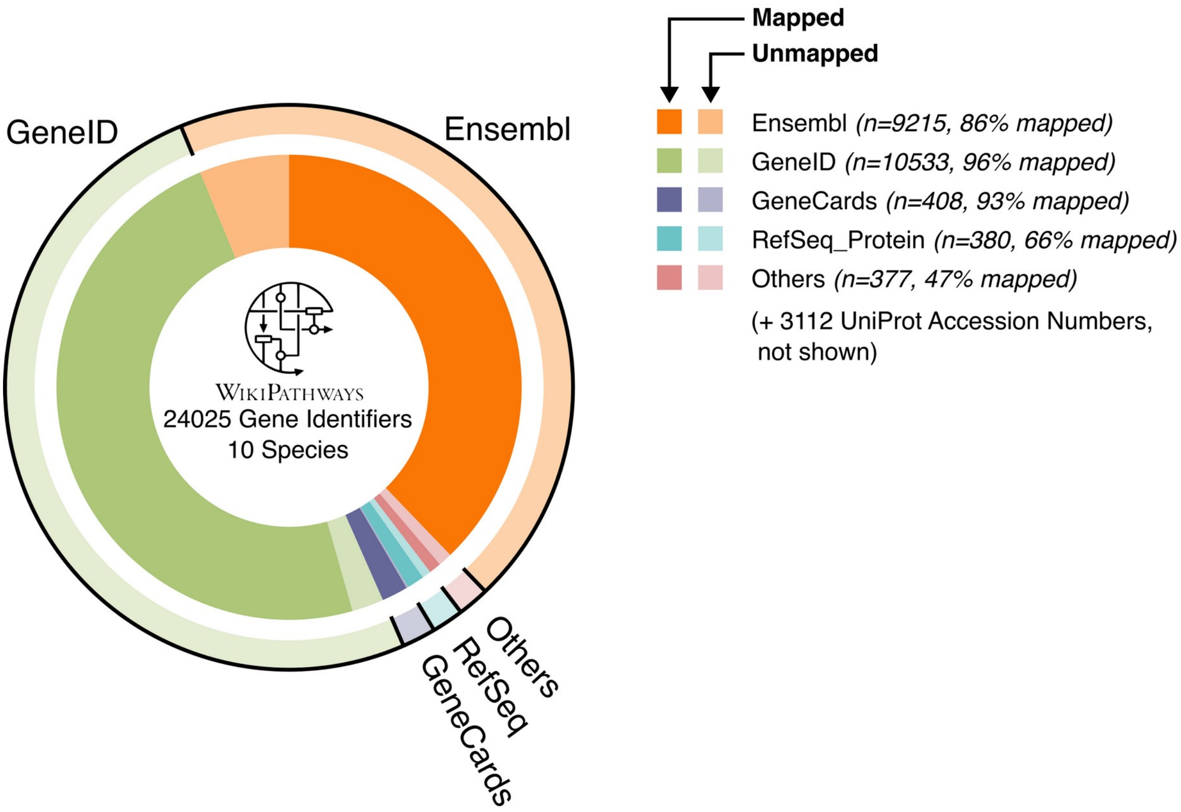


**Supplementary Figure 4. Gene Identifier Conversion for WikiPathways Diagrams.**

*WikiPathways uses 9 different gene identifier types in the diagrams processed in this study. The UniProt API was used to map them to UniProt Accession Numbers. Overall, 91% of gene identifiers (21956 of 24025) could be mapped (or were already in UniProt format). 'Ensembl' summarizes identifiers of type 'Ensembl' and 'Ensembl_Transcript'. 'Others' summarizes 'KEGG', 'EC', and 'HGNC' identifiers. Source data are provided as a Source Data file. The WikiPathways Logo is licensed under the CreativeCommons BY-SA license (https://creativecommons.org/licenses/by-sa/4.0/deed.en) by Alexander Pico (https://www.wikipathways.org/authors/AlexanderPico.html).*

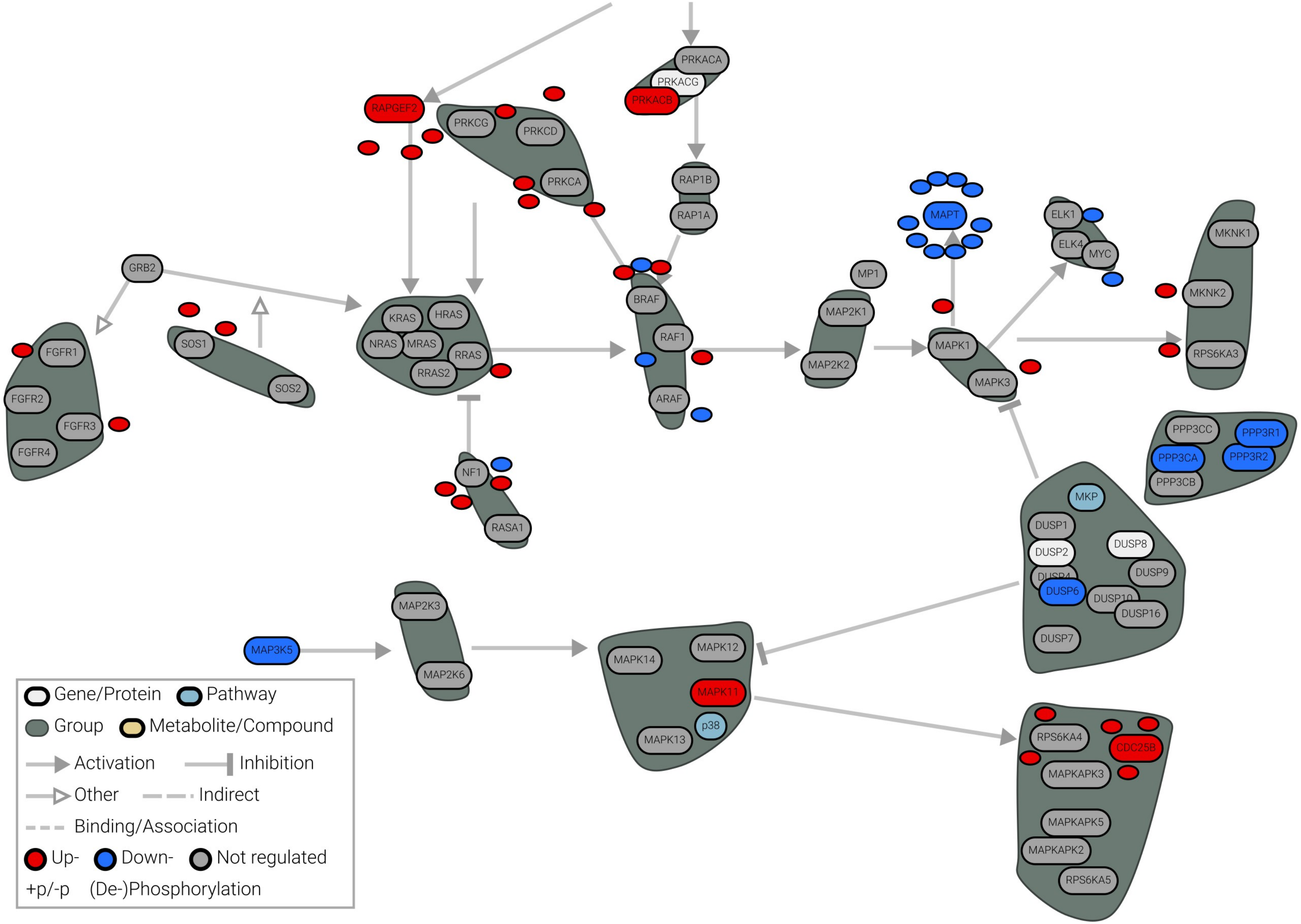

RAPGEF2
PRKCG
PRKCD
PRKCA
PRKACA
PRKACG
PRKACB
RAP1B
RAP1A
GRB2
FGFR1
FGFR2
FGFR3
FGFR4
SOS1
SOS2
KRAS
HRAS
NRAS
MRAS
RRAS
RRAS2
NF1
RASA1
BRAF
RAF1
ARAF
MP1
MAP2K1
MAP2K2
MAPT
MAPK1
MAPK3
ELK1
ELK4
MYC
MKNK1
MKNK2
RPS6KA3
PPP3CC
PPP3R1
PPP3CA
PPP3R2
PPP3CB
MKP
DUSP1
DUSP2
DUSP8
DUSP9
DUSP6
DUSP10
DUSP16
DUSP7
MAP3K5
MAP2K3
MAP2K6
MAPK14
MAPK12
MAPK11
p38
MAPK13
RPS6KA4
CDC25B
MAPKAPK3
MAPKAPK5
MAPKAPK2
RPS6KA5
Gene/Protein
Pathway
Group
Metabolite/Compound
Activation
Inhibition
Other
Indirect
Binding/Association
Up-
Down-
Not regulated
+p/-p (De-)Phosphorylation

**Supplementary Figure 5. Visualization of protein-level and peptide-level data in PTMNavigator.**

*Comparison of baseline proteomes and phosphoproteomes of the cell lines A204 and RDES projected onto the diagram 'MAPK Signaling - WP382'. Colors indicate whether expression is higher in A204 (red) or RDES (blue). Grey color indicates peptides/proteins that were not sufficiently different between the two cell lines (as thresholds, a difference of 3 was used for proteomic and 4 for phosphoproteomic data). White protein nodes indicate proteins that were not measured in the experiment. Dataset generated by Lee et al. Source data are provided as a Source Data file.*

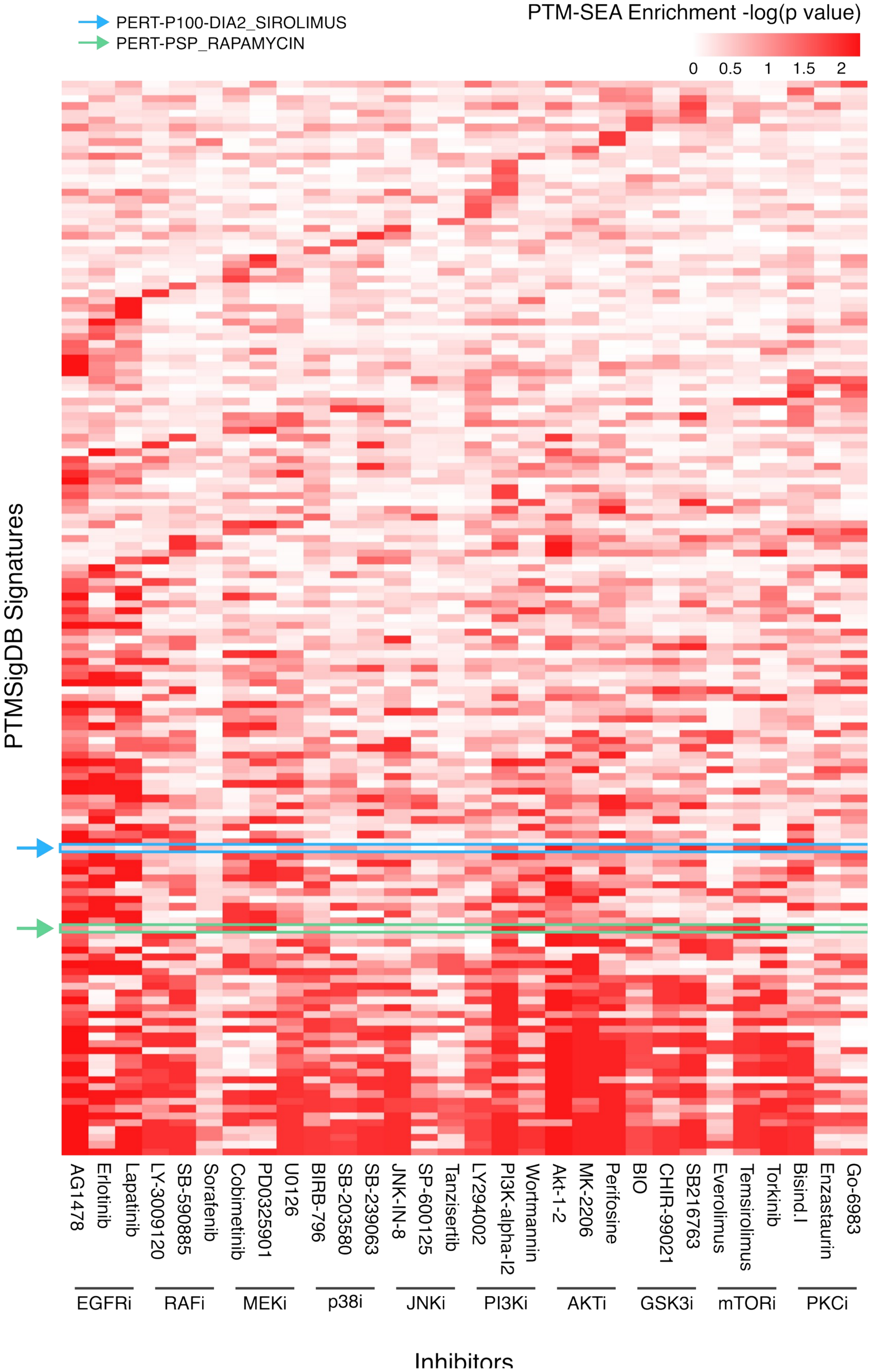
PERT-P100-DIA2_SIROLIMUS
PERT-PSP_RAPAMYCIN
PTM-SEA Enrichment -log(p value)
0 0.5 1 1.5 2
PTMSigDB Signatures
AG1478
Erlotinib
Lapatinib
LY-3009120
SB-590885
Sorafenib
Cobimetinib
PD0325901
U0126
BIRB-796
SB-203580
SB-239063
JNK-IN-8
SP-600125
Tanzisertib
LY294002
PI3K-alpha-I2
Wortmannin
Akt-1-2
MK-2206
Perifosine
BIO
CHIR-99021
SB216763
Everolimus
Temsirolimus
Torkinib
Bisind.I
Enzastaurin
Go-6983
EGFRi
RAFi
MEKi
p38i
JNKi
PI3Ki
AKTi
GSK3i
mTORi
PKCi
Inhibitors

**Supplementary Figure 6. Results of PTM-SEA analysis of the Bekker-Jensen dataset.**

*Shown are signatures with p-value<0.05, sorted by the number of datasets in which they were reported as enriched (larger numbers on the bottom). Inhibitors are grouped by their designated targets. Source data are provided as a Source Data file.*

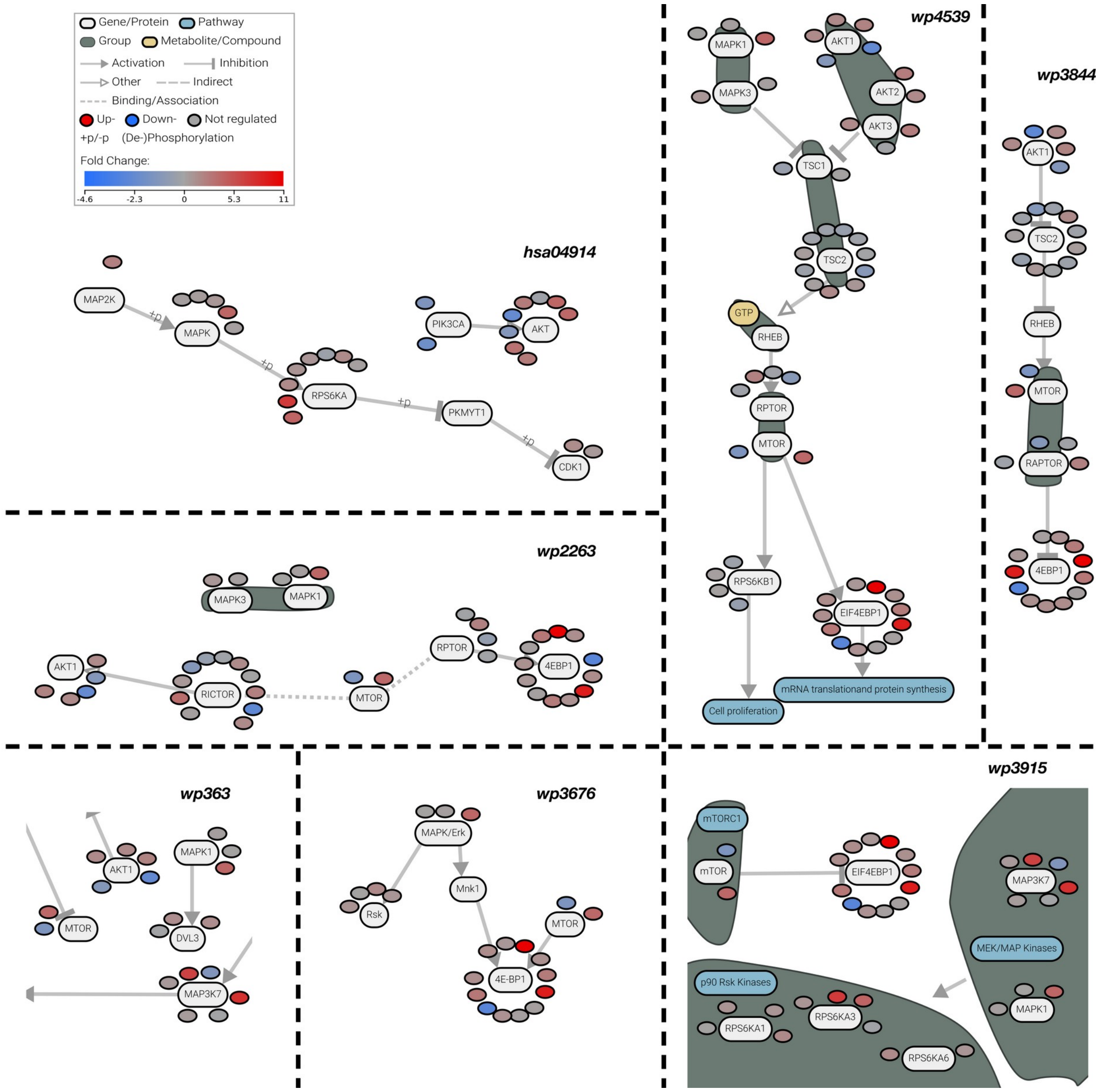


**Supplementary Figure 7. Common subnetworks of the high-scoring pathways in the Go-6983 dataset.**

*Edited Screenshots from PTMNavigator showing excerpts from 7 different canonical pathway diagrams. All of these pathways showed significant enrichment in the gene-centric-redundant ssGSEA. PTM nodes are colored according to their fold change. Edges labeled with '+p' indicate a phosphorylation of the target protein. Source data are provided as a Source Data file.*

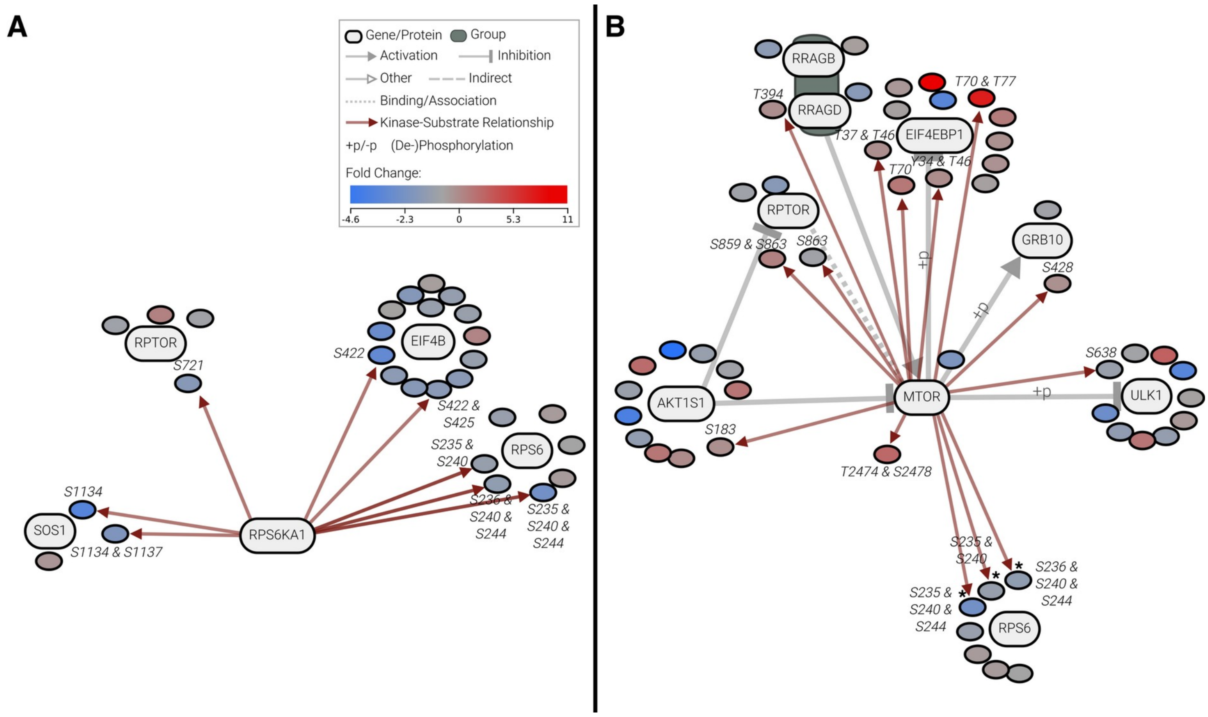


**Supplementary Figure 8. PTMNavigator makes PTM-SEA and kinase activity scores transparent.**

*Edited Screenshots from PTMNavigator showing excerpts from the projection of the Go-6983 dataset onto the pathway 'mTOR Signaling Pathway - hsa04150'. Shown are the proteins phosphorylated by RPS6KA1 (**A**) and mTOR (**B**). Red arrows indicate kinase-substrate annotations from PhosphoSitePlus. Both PTM-SEA and KSEA reported a downregulation of RPS6KA1 activity as well as an upregulation of mTOR activity. The visualizations of PTMNavigator confirm this, with all annotated RPS6KA1 substrates in the diagram showing reduced expression, whereas most mTOR substrates showed increased expression. Notably, the sites on RPS6 (indicated by asterisks '*') are not exclusively associated with mTOR, but are also substrates of RPS6KA1, RPS6KA3, and PRKCD. Edges labeled with '+p' indicate a phosphorylation of the target protein. Source data are provided as a Source Data file.*

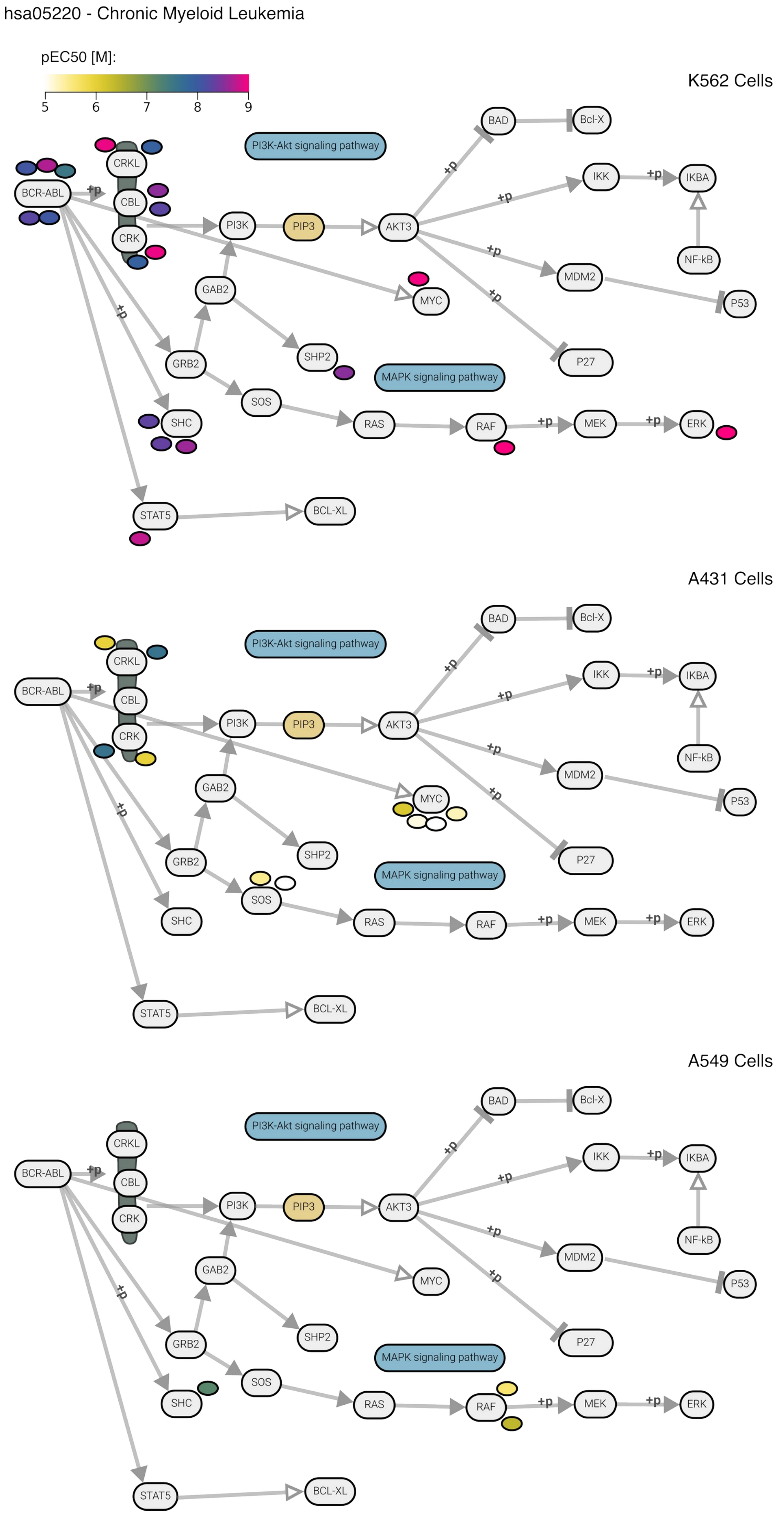

hsa05220 - Chronic Myeloid Leukemia
pEC50 [M]:
5
6
7
8
9
K562 Cells
A431 Cells
A549 Cells
PI3K-Akt signaling pathway
MAPK signaling pathway
BCR-ABL
CRKL
CBL
CRK
PI3K
PIP3
AKT3
GAB2
GRB2
SHC
SOS
SHP2
RAS
RAF
MEK
ERK
STAT5
BCL-XL
MYC
BAD
Bcl-X
IKK
IKBA
NF-kB
MDM2
P53
P27
+p

**Supplementary Figure 9. Comparison of dose-dependent Dasatinib treatment in three different cell lines.**

*Edited screenshots from PTMNavigator. Projection of data from Zecha et al. onto the KEGG diagram 'hsa05220 – Chronic Myeloid Leukemia'. Edges labeled with '+p' indicate a phosphorylation of the target protein. Top: K562 lymphoblast cells. Middle: A431 epidermoid carcinoma epithelial cells. Bottom: A549 lung adenocarcinoma cells. Source data are provided as a Source Data file.*

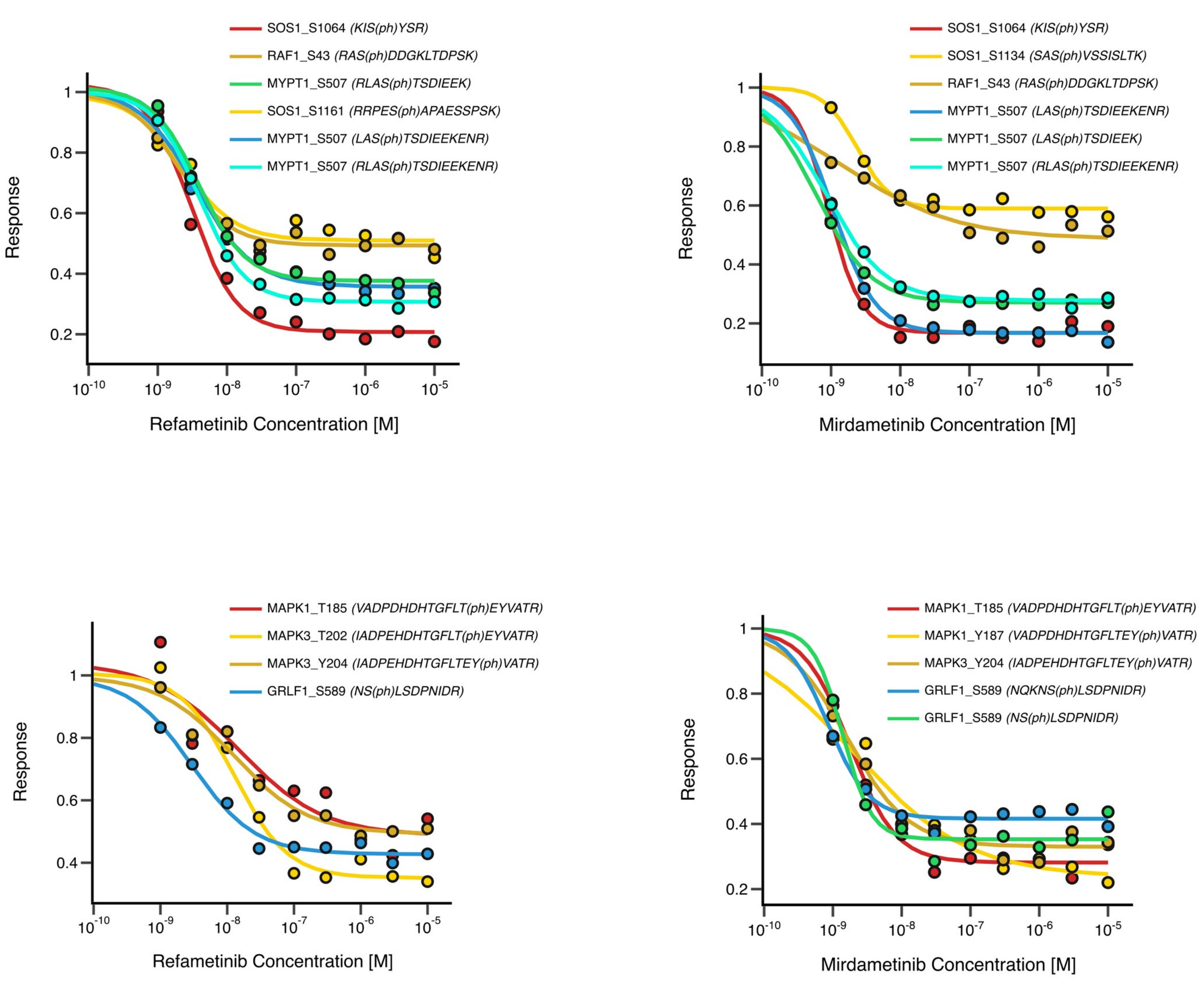


**Supplementary Figure 10. decryptM curves from the Refametinib and Mirdametinib datasets.**

*Source data are provided as a Source Data file.*
