# Supplementary material for: PTMNavigator: interactive visualization of differentially regulated post-translational modifications in cellular signaling pathways
Source: Nat Commun. 2025 Jan 8;16:510. doi: 10.1038/s41467-024-55533-y (PMC11711753; doi:10.1038/s41467-024-55533-y)
Supplement: Supplementary file 3 — Description of Additional Supplementary Files [file 41467_2024_55533_MOESM3_ESM.docx]

File Name: Supplementary Data 1

Description: Input examples for PTMNavigator.
